# Efficacy of repetitive peripheral magnetic stimulation on upper limb motor function after stroke: a systematic review and meta-analysis of randomized controlled trials

**DOI:** 10.3389/fneur.2025.1612490

**Published:** 2025-09-18

**Authors:** Junxia Liu, Meirong Zhu, Xiaoyan Liu, Weiju Tang, Yunan Xiang, Yulei Xie, Yinxu Wang

**Affiliations:** ^1^First People’s Hospital of Longquanyi District, Chengdu, China; ^2^Department of Rehabilitation Medicine, Affiliated Hospital of North Sichuan Medical College, Nanchong, China

**Keywords:** repetitive peripheral magnetic stimulation, stroke rehabilitation, upper limb motor function, systematic review, meta-analysis, stroke

## Abstract

**Background:**

Approximately 50–70% of stroke survivors are left with varying degrees of limb paralysis, severely affecting their ability to perform daily activities and engage in rehabilitation. Although conventional rehabilitation interventions, such as task-oriented training and transcranial magnetic stimulation (TMS), have been widely utilized, their efficacy has been constrained by individual differences and limitations in neuroplastic activation. Repetitive peripheral magnetic stimulation (rPMS), a novel non-invasive neuromodulation technique, directly targets peripheral nerves and muscles to potentially facilitate the remodeling of motor pathways. There is a lack of evaluation regarding the effectiveness of rPMS for improving upper limb motor function and spasticity in stroke patients.

**Methods:**

Randomized controlled trials examining the effects of rPMS in post-stroke patients, published up to 20 February 2025, were searched in PubMed, Embase, the Cochrane Library, and the Web of Science. Methodological quality was evaluated using the Cochrane Collaboration tool. Meta-analyses were performed using RevMan (version 5.4). The Grading of Recommendations, Assessment, Development and Evaluation (GRADE) method was used to assess the quality of evidence.

**Results:**

A total of 12 studies involving 492 patients were included. The results of the meta-analysis indicated that, compared to the control group, the subgroup analyses based on disease stage, stimulation frequency, coil type, stimulation duration, and stimulation intensity showed significant improvements, supported by high-quality evidence. The pooled standardized mean differences (SMDs) were as follows: disease stage, SMD = 0.69 (*p* = 0.006); stimulation frequency, SMD = 0.58 (*p* = 0.004); coil type, SMD = 0.82 (*p* = 0.001); stimulation duration, SMD = 0.62 (*p* = 0.004); and stimulation intensity, SMD = 0.79 (*p* = 0.002). In addition, rPMS significantly improved patients’ ability to live independently (SMD = 0.66, *p* <0.0001), supported by moderate-quality evidence. However, rPMS did not demonstrate a significant effect in reducing spasticity [mean difference (MD) = 0.25, *p* = 0.20], with this finding supported by low-quality evidence.

**Systematic review registration:**

rPMS improved upper limb motor function, activities of daily living (ADL), and self-care abilities in post-stroke patients, with good acceptability and only mild adverse reactions. Its effect on spasticity was not significant.

**Systematic review registration:**

www.crd.york.ac.uk/prospero/, CRD420250637455.

## 1 Introduction

Globally, stroke is the second leading cause of death (11.6% of total deaths) and the third leading cause of disability (5.7% of total DALYs) (1). Stroke could cause a series of complications, such as dysphagia, consciousness dysfunction, limb motor dysfunction, and cognitive dysfunction (2), and approximately 80% of survivors after stroke are left with upper limb dysfunction (3). At present, the effectiveness of traditional treatments for upper limb motor dysfunction after stroke is limited by poor patient compliance, insufficient activation of central plasticity, and a narrow rehabilitation period. These limitations seriously affect patients’ ability to perform daily living and normal activities, causing serious challenges and losses for survivors and their families in terms of quality of life and economy. Repetitive peripheral magnetic stimulation (rPMS), as a non-invasive neuromodulation technique, can affect the excitability and inhibition of the motor cortex by penetrating the deep structures of the brain through painless stimulation. This promotes the plasticity of the motor cortex and further causes changes in brain function (4). Numerous studies have found that rPMS can significantly improve upper limb motor function and daily living abilities after stroke (5–7), providing a mechanism and empirical evidence for exploring its application in the rehabilitation of the upper limb. A growing number of meta-analyses with small sample sizes and non-uniform parameters and outcome indicators have demonstrated the positive effects of rPMS on motor function after stroke (5, 8, 9), limiting the comparability of the assessments of efficacy. This study aimed to comprehensively evaluate the effectiveness of rPMS on upper limb dysfunction after stroke and to provide the latest evidence to guide clinical practice.

## 2 Materials and methods

This study was registered with PROSPERO under registration number CRD420250637455. It was conducted following Preferred Reporting Items for Systematic Reviews and Meta-Analyses (PRISMA) guidelines (10).

### 2.1 Search strategies

The databases, including Embase, PubMed, the Cochrane Library, the Web of Science, and China National Knowledge Infrastructure (CNKI), were searched to identify studies on the effect of rPMS on post-stroke upper limb motor dysfunction up to 20 February 2025. The English keywords for the database searches included “stroke,” “cerebrovascular accident,” “upper extremity,” “motor function,” “motor performance,” “repetitive peripheral magnetic stimulation,” “peripheral magnetic stimulation,” “magnetic field,” “rPMS,” and “PMS.” The reference lists of the identified articles were checked for potential studies. The detailed search strategies for each database are provided in Supplementary Table S1.

### 2.2 Inclusion and exclusion criteria

Two reviewers independently conducted the literature screening. Disagreements were recorded and resolved through discussions with a third reviewer. The inclusion criteria were as follows: (1) randomized controlled trial (RCT) studies; (2) studies involving participants who experienced a first-time stroke with upper limb motor dysfunction, confirmed by magnetic resonance imaging or computed tomography; and (3) studies in which the experimental group received rPMS treatment in addition to the control group’s intervention, which could be a placebo, sham, or routine rehabilitation. The exclusion criteria were as follows: (1) animal experiments or studies including healthy volunteers; (2) studies without the target outcome measures; (3) studies for which the full text was not available; and (4) studies lacking complete outcome data. For studies with overlapping data, those with larger or more complete datasets were prioritized. Both published and unpublished studies were considered, and authors were contacted if additional details not reported in the articles were needed.

### 2.3 Risk of Bias and quality of outcomes assessment

Two reviewers independently evaluated the methodological quality of all included studies. A third reviewer recorded and resolved any disagreements. The Cochrane Collaboration Tool was utilized to assess the risk of bias for each RCT, including adequacy of sequence generation, concealment of allocation, blinding of participants and personnel, blinding of result evaluators, incomplete result data, and selective reporting (11, 12). The Grading of Recommendations, Assessment, Development and Evaluation (GRADE) guidelines for systematic reviews were followed to assess the quality of outcomes (13).

### 2.4 Data extraction

All included studies were conducted by two independent reviewers. If there was any disagreement, a third reviewer made the final decision. The following data were extracted from the included studies: basic information (study authors, year of publication), participant characteristics (age, time post-stroke, and sample size), rPMS parameters (site, frequency, intensity, and regimen of stimulation), outcome indicators of upper limb motor function, and activities of daily living (ADL).

### 2.5 Outcome indicators

The primary outcome included the Fugl-Meyer Assessment of the Upper Extremity (FMA-UE). Secondary outcome indicators for efficacy included the Modified Ashworth Scale (MAS), Functional Independence Measure (FIM), and Modified Barthel Index (MBI), as well as dropout rate and adverse effects.

As the first quantitative tool developed to assess the recovery of sensory and motor function after stroke, the FMA has been extensively tested in clinical settings and proven to be both feasible and effective in stroke. The scale is divided into five domains, namely motor function, sensory function, balance, joint range of motion, and joint pain. The FMA-UE evaluates the movement, coordination, and reflexes of the upper limbs (the shoulders, elbows, forearms, wrists, and hands), with its score ranging from 0 (hemiplegia) to 66 (normal motor performance) (14). The MAS is the most commonly used clinical tool for assessing muscle tension and spasticity. It is graded from 0 to 4 (0, 1, 1+, 2, 3, 4), where 0 indicates no resistance and 4 indicates limb stiffness during flexion or extension (15). The FIM, as a scale for evaluating functional independence, assesses 18 kinds of activities of daily living on a 7-point scale, ranging from 1 (completely dependent) to 7 (unassisted independent) (16). The MBI is a five-point scale used to measure activities of daily living, and Tomoko Ohura et al. affirmed its reliability and validity after stroke (17). The outcomes post-intervention from the follow-up phase were selected for meta-analysis if they were reported at multiple time points.

### 2.6 Statistical analyses

All statistical analyses were performed using RevMan 5.4 software (The Nordic Cochrane Centre, Cochrane Collaboration, Copenhagen, Denmark). Heterogeneity among studies was assessed using the chi-squared (Chi^2^) test and the *I*^2^ statistic. A fixed effects model was applied when heterogeneity was low (*I*^2^ <50%), whereas a random effects model was used when heterogeneity was substantial (*I*^2^ ≥ 50%). Dichotomous data were presented as risk ratios (RRs) with 95% confidence intervals (CIs), and continuous data were expressed as mean differences (MDs) or standardized mean differences (SMDs) with 95% CIs. Subgroup meta-analyses of the primary outcome were conducted based on predefined variables, such as disease stage, stimulation frequency, coil type, stimulation duration, and stimulation intensity. Combined effect sizes were calculated within each subgroup, and differences between the subgroups were compared.

## 3 Results

### 3.1 Search and selection of studies

**Figure 1:**
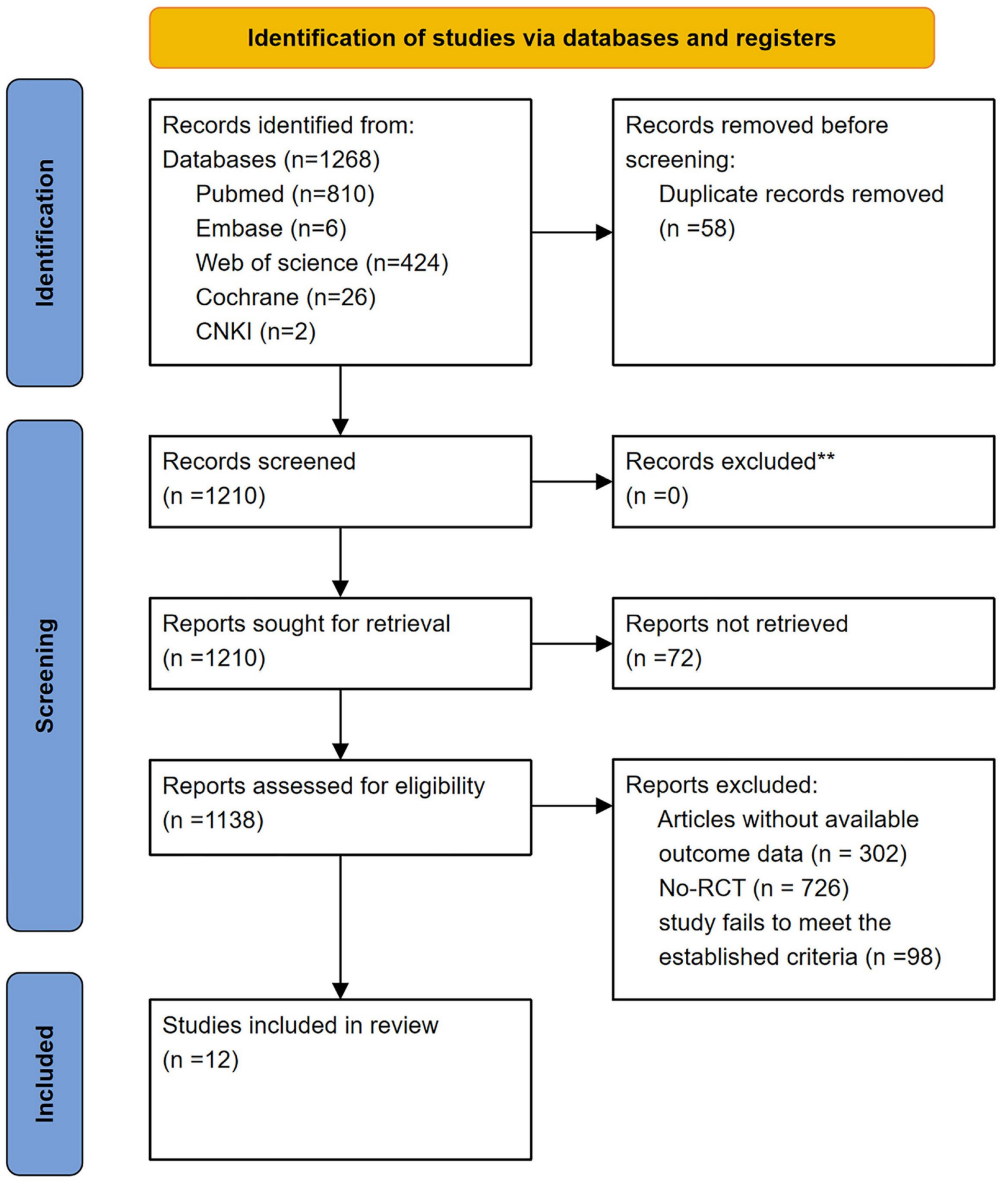
PRISMA 2020 flow diagram for new systematic reviews including searches of databases and registers only.

The selection process of this study is shown in Figure 1. A total of 1,268 potentially relevant studies were screened from four English databases and CNKI using relevant search strategies. Then, 58 duplicates were removed, and 72 studies were excluded because they were too old to obtain the full text. An additional 1,028 studies were removed after screening titles and abstracts. Finally, after reviewing the full texts of the remaining 110 articles, a total of 12 studies were finally included.

### 3.2 Characteristics of the included studies

A total of 12 studies with 484 participants were included in this study. The characteristics of the included studies are presented in Table 1. The site, frequency, treatment intensity, number of pulses, on–off ratio, treatment duration, and coil type of rPMS stimulation differed between these studies.

**Table 1:** Characteristics of the included studies.

Study	Participants	Sex	Age (mean/median)	Time post-stroke (mean/median)	Targeted points	Frequency intensity pulses	On/off (s)	Treatment time	Coil type	CG intervention	Additional intervention	Outcomes
Krewer et al. (24)	63	25/38	EG: 55.00	EG: 26.00 weeks	Extensors and flexors of the upper arm	25 Hz, above 10% MCT, 5000	1/2	20 min, 2 times/day, 2 weeks	Butterfly	Sham	OT	FMA-UE, MTS, BI
			CG: 54.00	CG: 37.00 weeks								
Yang et al. (27)	30	23/7	EG: 63.67	EG: 13.87 days	Supraspinatus, deltoid muscles	5 Hz, 100% RMT	NA	40 min, 4 weeks	Figure-of-eight	Conventional rehabilitation	NA	FMA-UE
			CG: 67.20	CG: 15.47 days								
Chen et al. (18)	32	23/9	EG: 49.00	EG: 37.40 month	From shoulder adductors to extensors, elbow flexors to extensors, wrist flexors to extensors	5 Hz/20 Hz, 750/5100	3/1	30 min, one time	Parabola	Sham	NA	FMA-UE, MAS, MTS
			CG: 45.60	CG: 22.80 month			1.5/1					
Obayashi and Takahashi (20)	19	13/6	EG: 64.30	EG: 9.20 days	Extensor digitorum communis, extensor carpi radialis, flexor digitorum superficialis, triceps brachii, biceps brachii, anterior or middle head of deltoid	30 Hz, 70% MSO	2/2	15–20 min, until transfer	Round	Standard care	Standard care	FMA-UE, WMFT, FAS, BBT
			CG: 72.30	CG: 5.80 days								
Jiang et al. (6)	44	27/17	EG: 54.60	EG: 13.81 weeks	Triceps brachii	20 Hz, 15–30% MSO, 2400	0.5/2	20 min, 2 weeks	Round	Untreated	PT	FMA-UE
			CG: 56.09	CG: 14.45 weeks								
Ke et al. (25)	30	14/12	EG: 58	EG: 17 days	Axilla	20 Hz, 40–60% MSO, 1800	1/19	30 min, 10 days	Figure-of-eight	Sham	Conventional treatments	FMA-UE, MRC
			CG: 56	CG: 16 days								
El Nahas et al. (23)	42	27/9	EG: 47.88	NA	Biceps brachii, wrist/finger flexor group	50 Hz, above MCT, 600	2/8	1,600 s, 8 days	Figure-of-eight	Sham	NA	MAS
			CG: 41.60									
Fawaz et al. (19)	80	56/24	57.33	NA	Shoulder abductors, elbow extensors, wrist extensors, supinator muscle	30 Hz, above 10% MCT, 4500	5/1	30 min, 3 weeks	Round, butterfly	Sham	OT	FMA-UE, FIM
Wu et al. (9)	30	27/3	EG: 57.00	EG: 31.89 days	Cervical nerve root	10 Hz, 80% RMT	1/5	1,000 s, 3 weeks	Round, figure-of-eight	Conventional rehabilitation	PT, OT	FMA-UE, WMFT, BBT
			CG: 55.33	CG: 41.58 days								
Chang et al. (22)	28	15/13	EG: 51.40	NA	Arm	5 Hz, individually adjusted	2/8	2 weeks	Figure-of-eight	Sham	PT, OT, iTBS	FMA-UE, ARAT, FIM
			CG: 55.60									
Xie et al. (26)	40	29/10	EG: 61.60	EG: 33.05 days	Radial nerve (superficial course above the elbow joint)	25 Hz, 120% RMT, 5000	5/15	15 min, 2 weeks	Figure-of-eight	Conventional rehabilitation	NA	iEMC, RMS, MF, FMA-UE, ARAT, MBI, MAS
			CG: 64.00	CG: 30.25 days								
Fujimura (41)	46	31/45	EG: 69.00	EG: 34.00 days	Shoulder, elbow, forearm, wrist, hand	30 Hz, 0.65–0.9Tesla, 6,000	2/3	17 min, 6 weeks	NA	Conventional rehabilitation	NA	FMA-UE, AHI
			CG: 61.00	CG: 41.00 days								

EG, experimental group; CG, control group; MCT, muscle contraction threshold; RMT, resting motion threshold; MSO, maximal stimulator output; OT, occupational therapy; PT, physical therapy; iTBS, intermittent theta burst stimulation; FMA-UE, Fugl-Meyer Assessment of the Upper Extremity; MTS, Modified Tardieu Scale; MBI (BI), Modified Barthel Index (Barthel Index); MAS, Modified Ashworth Scale; WMFT, wolf motor function test; FAS, Functional Ability Scale; BBT, Box and Block Test; MRC, medical research council scale; FIM, Functional Independence Measure; ARAT, Action Research Arm Test; iEMC, integrated electromyography; RMS, root mean square; MF, median frequency; AHI, acromiohumeral interval.

A total of eight studies (18–24) stimulated more than two groups of upper limb muscles, with one study (6) stimulating only the triceps brachii muscle and another (25) stimulating the axilla of the affected arm. Furthermore, two studies targeted nerves: one at the cervical nerve root (9) and the other at the radial nerve above the elbow joint (26). The frequency of stimulation was ≤20 Hz in six studies (6, 9, 18, 22, 25, 27) and >20 Hz in six studies (19–21, 23, 24, 26). In addition, six studies (19, 21, 23, 24, 26, 27) determined the intensity of treatment based on the percentage of the maximum output value of the treatment apparatus, and four studies (6, 9, 20, 25) determined it based on the intensity of movement occurring in the wrist at rest. Moreover, one study (22) individualized treatment for patients, while one study (18) did not mention details. In total, five studies had a daily stimulation time of >20 min (18, 19, 23, 25, 27), six studies had a daily stimulation time of ≤20 min (6, 9, 20, 21, 24, 26), and one study (22) did not report the duration. There were seven studies (6, 18, 22–26) with a treatment duration of ≤2 weeks, four studies (9, 19, 21, 27) with a duration of >2 weeks, and one study (20) determined the duration based on the transfer time to the hospital. Six studies (18, 19, 22–25) used sham stimulation as the control, four studies (9, 21, 26, 27) used conventional rehabilitation, one study (20) used standard care, and one study (6) had no treatment in the control group.

### 3.3 Research quality

**Figure 2:**
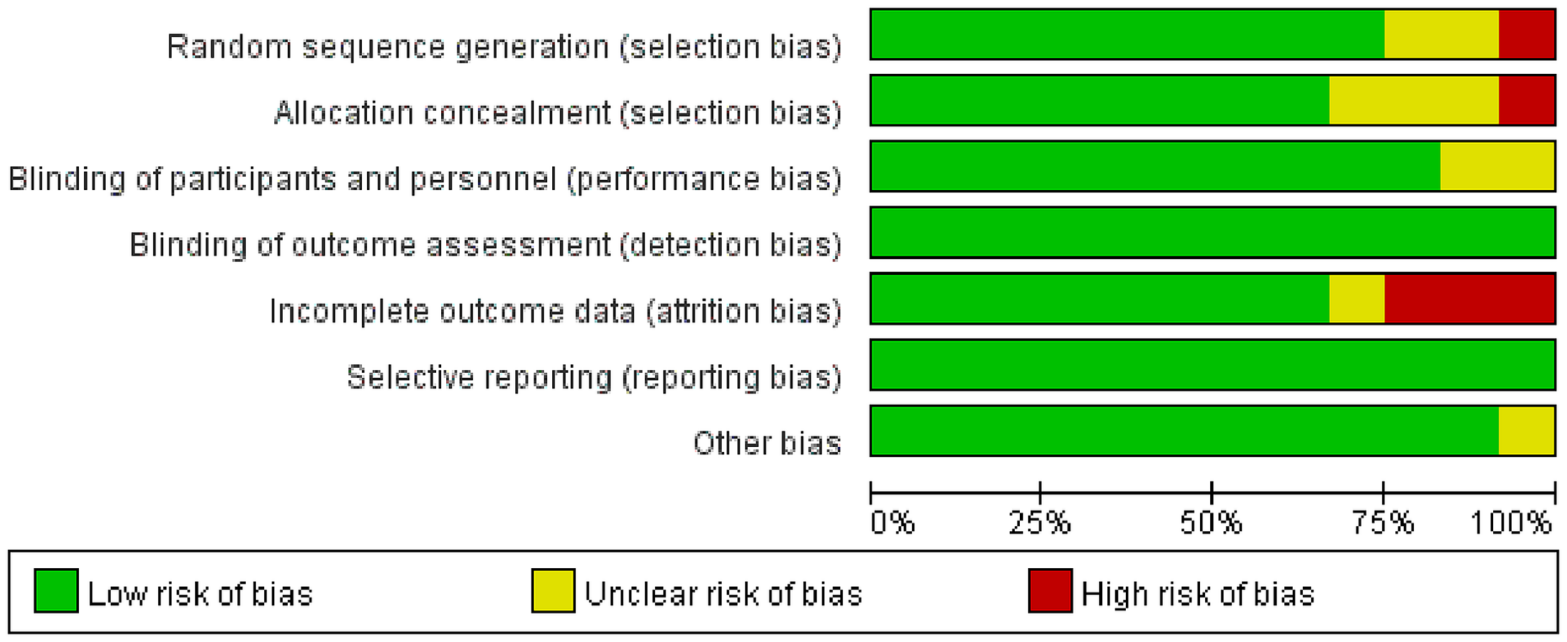
Summary of the risk of bias assessment.

**Figure 3:**
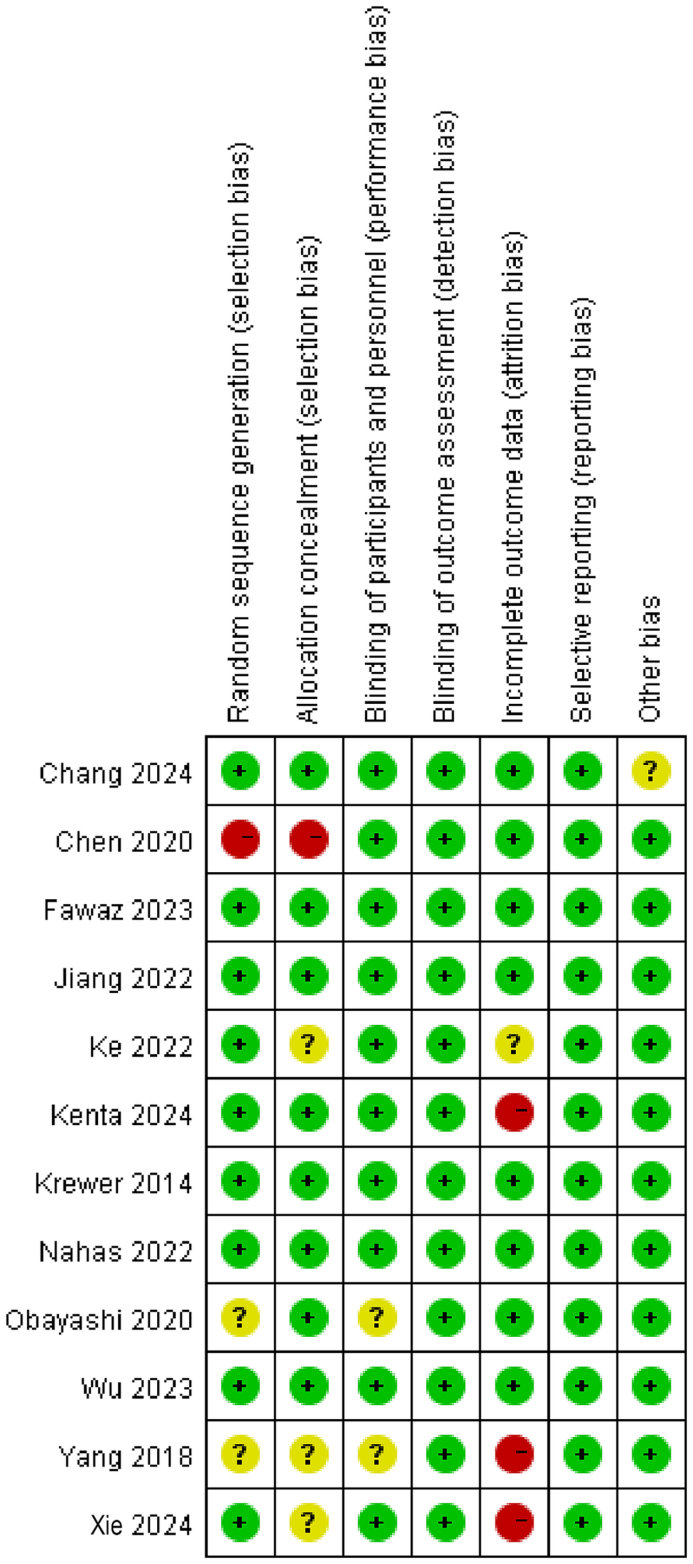
Forest plot of the risk of bias assessment.

As shown in Figures 2, 3, the 12 included studies showed low risk of bias in terms of blinding of outcome assessment (detection bias) and selective reporting (reporting bias), indicating that these studies were relatively standardized in their design and execution. However, a small number of high-risk biases were identified in random sequence generation (selection bias), blinding of participants and personnel (performance bias), and incomplete outcome data (attrition bias), indicating potential statistical errors in these areas. In addition, five studies (6, 9, 19, 23, 24) were assessed as low risk and high quality in terms of methodological quality. There were four studies (18, 21, 26, 27) with high-risk indicators. Among them, Chen et al.’s study (18) showed a high risk of bias in random allocation and allocation concealment, which likely led to selection bias because of non-random allocation. In total, three studies (21, 26, 27) had a high risk of loss of follow-up bias in terms of data integrity. In addition, three studies (20, 22, 25) had unknown risks on a small number of indicators and showed low levels of bias. Overall, the included studies showed high methodological quality and a high evidence level.

### 3.4 Meta-analysis of the FMA-UE

**Figure 4:**
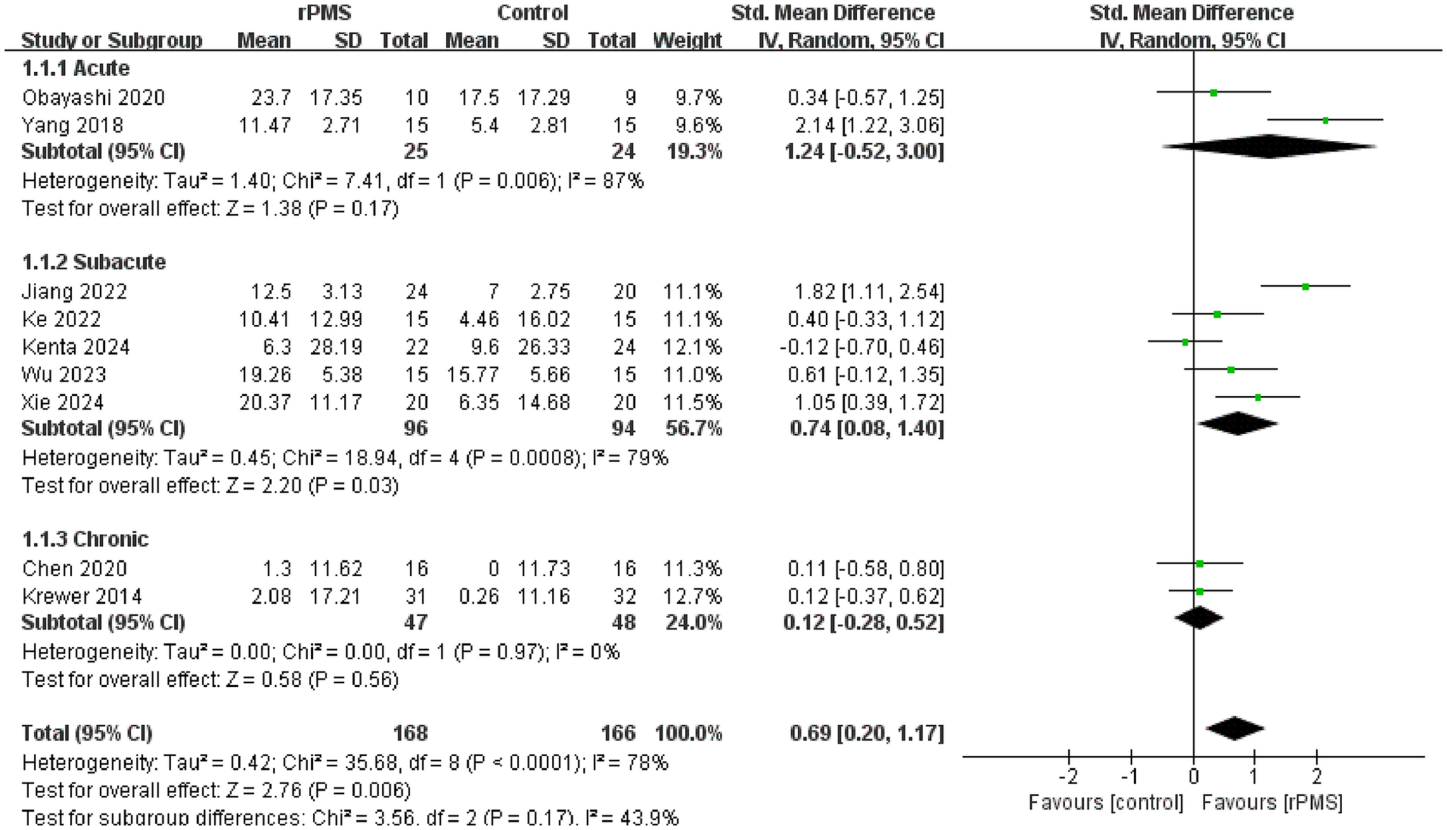
Forest plot of the effects of rPMS on upper limb motor function in stroke patients at different disease stages.

**Figure 5:**
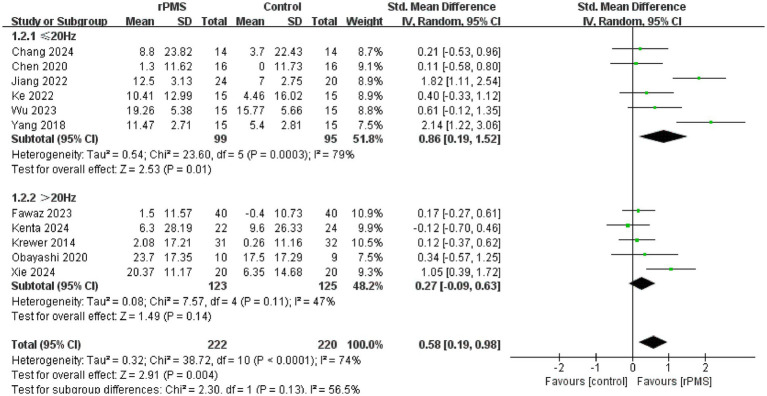
Forest plot of the effects of rPMS on upper limb motor function in stroke patients at different stimulation frequencies.

**Figure 6:**
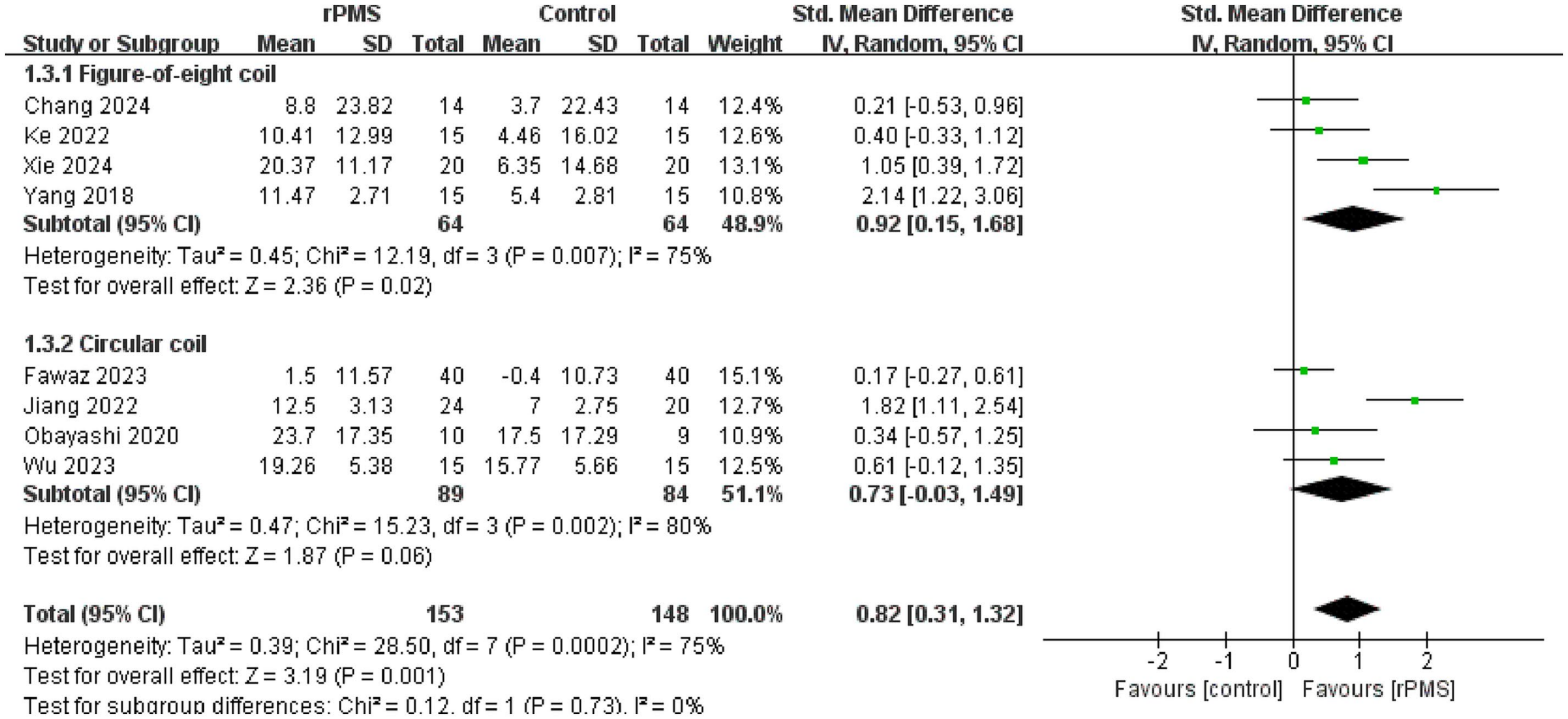
Forest plot of the effects of rPMS on upper limb motor function in stroke patients using different coil types.

**Figure 7:**
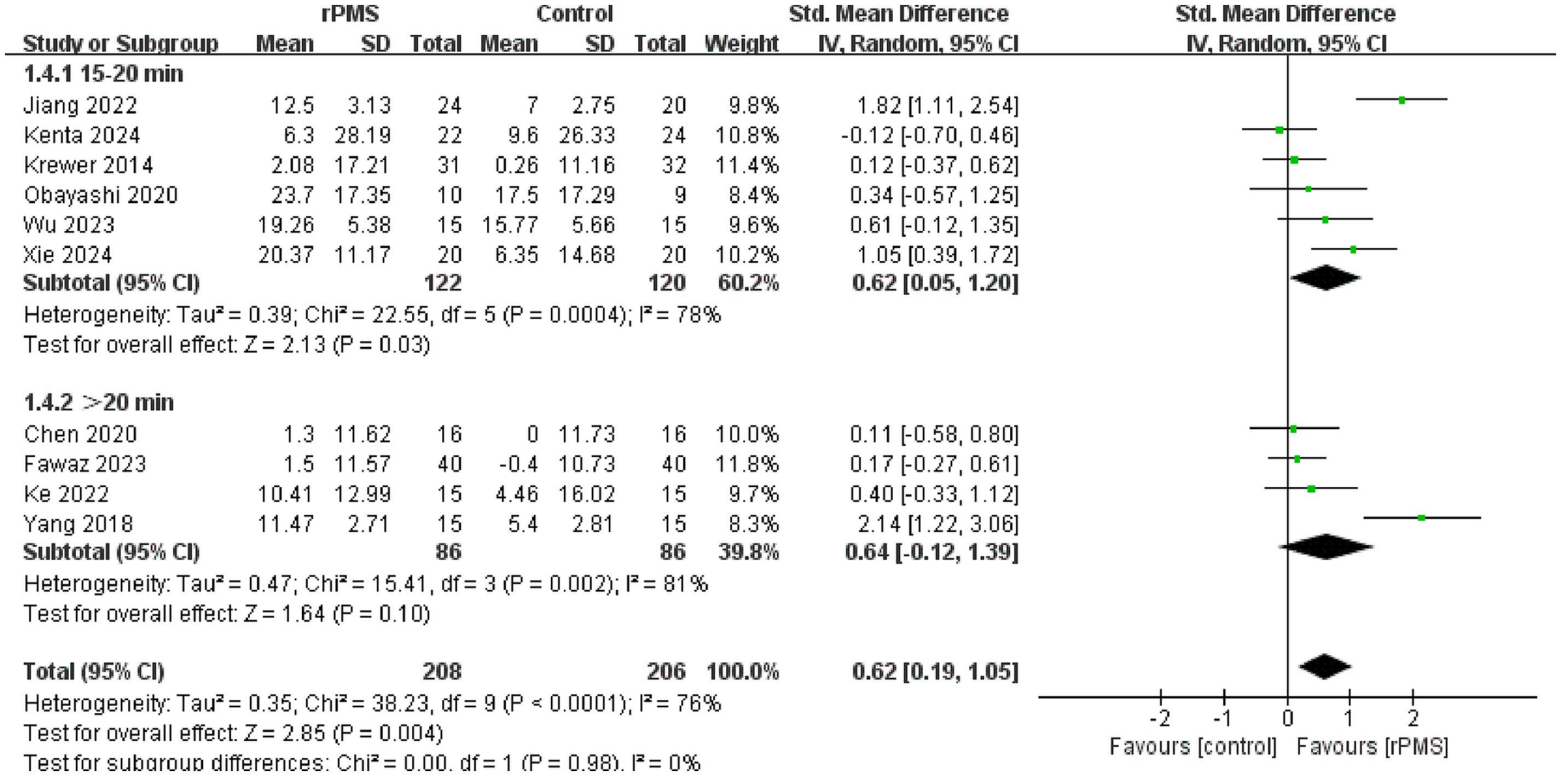
Forest plot of the effects of rPMS on upper limb motor function in stroke patients with different stimulation durations.

**Figure 8:**
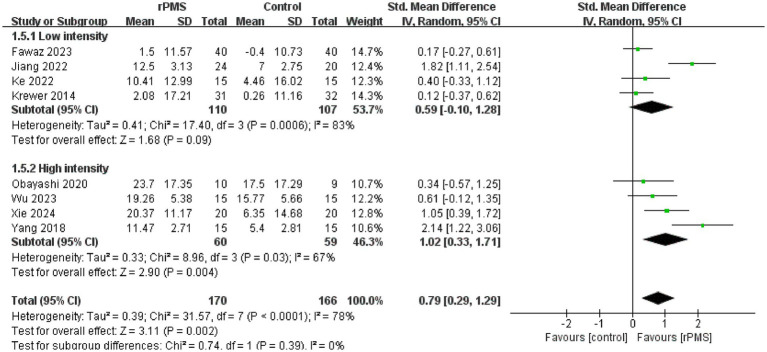
Forest plot of the effects of rPMS on upper limb motor function in stroke patients with different stimulation intensities.

Furthermore, 11 studies (6, 9, 18–22, 24–27), involving a total of 442 participants, evaluated the effects of rPMS on the FMA-UE. Subgroup analyses were conducted based on disease stage, stimulation frequency, coil type, stimulation duration, and stimulation intensity. The meta-analysis conducted using a random effects model showed the following pooled SMDs: disease stage, SMD = 0.69 (95% CI 0.20–1.17, *p* = 0.006, *I*^2^ = 78%) (Figure 4); stimulation frequency, SMD = 0.58 (95% CI 0.19–0.98, *p* = 0.004, *I*^2^ = 74%) (Figure 5); coil type, SMD = 0.82 (95% CI 0.31–1.32, *p* = 0.001, *I*^2^ = 75%) (Figure 6); stimulation duration, SMD = 0.62 (95% CI 0.19–1.05, *p* = 0.004, *I*^2^ = 76%) (Figure 7); and stimulation intensity, SMD = 0.79 (95% CI 0.29–1.29, *p* = 0.002, *I*^2^ = 78%) (Figure 8).

In the disease stage subgroup, rPMS significantly improved upper limb motor function in patients during the subacute phase (SMD = 0.74, 95% CI 0.08–1.40, *p* = 0.03), while no significant effects were observed in acute-phase (SMD = 1.24, 95% CI −0.52–3.00, *p* = 0.17) or chronic-phase patients (SMD = 0.12, 95% CI −0.28–0.52, *p* = 0.56) (Figure 4). Regarding stimulation frequency, the ≤20 Hz subgroup showed significant improvement (SMD = 0.86, 95% CI 0.19–1.52, *p* = 0.01), whereas the >20 Hz subgroup did not demonstrate significant effects (SMD = 0.27, 95% CI −0.09–0.63, *p* = 0.14) (Figure 5). In terms of coil type, the figure-eight coil subgroup significantly enhanced FMA-UE scores (SMD = 0.92, 95% CI 0.15–1.68, *p* = 0.02), while the circular coil subgroup did not reach statistical significance (SMD = 0.73, 95% CI −0.03–1.49, *p* = 0.06) (Figure 6). Stimulation durations of 15–20 min showed significant effects (SMD = 0.62, 95% CI 0.05–1.20, *p* = 0.03, I^2^ = 78%), whereas durations over 20 min did not show significant differences (SMD = 0.64, 95% CI −0.12–1.39, *p* = 0.10) (Figure 7). For stimulation intensity, the high-intensity subgroup demonstrated significant effects (SMD = 1.02, 95% CI 0.33–1.71, *p* = 0.004), while the low-intensity subgroup did not reach statistical significance (SMD = 0.59, 95% CI −0.10–1.28, *p* = 0.09) (Figure 8).

### 3.5 Analysis of the MAS

**Figure 9:**
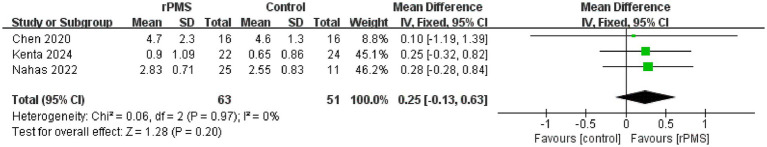
Forest plot of the effects of rPMS on spasticity in stroke patients with upper limb motor impairments.

A total of three studies (18, 21, 23), involving 114 participants, discussed the influence of rPMS on the MAS. The fixed effects analysis showed no significant improvement in upper limb spasticity compared to the control group (MD = 0.25, 95% CI −0.13−0.63, *p* = 0.20 > 0.05) (Figure 9).

### 3.6 Analysis of the FIM and MBI

**Figure 10:**
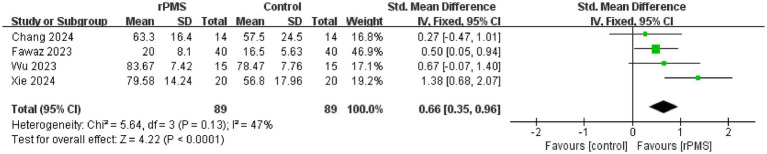
Forest plot of the effects of rPMS on activities of daily living in stroke patients with upper limb motor impairments.

A total of four studies (9, 19, 22, 26), involving 178 participants, discussed the FIM (19, 22) and MBI (9, 26). The fixed effects analysis showed that rPMS significantly improved patients’ ability to perform ADLs compared to the control group (SMD = 0.66, 95% CI 0.35–0.96, *p* < 0.0001) (Figure 10).

### 3.7 Meta-analysis of the dropout rate and adverse events

**Figure 11:**
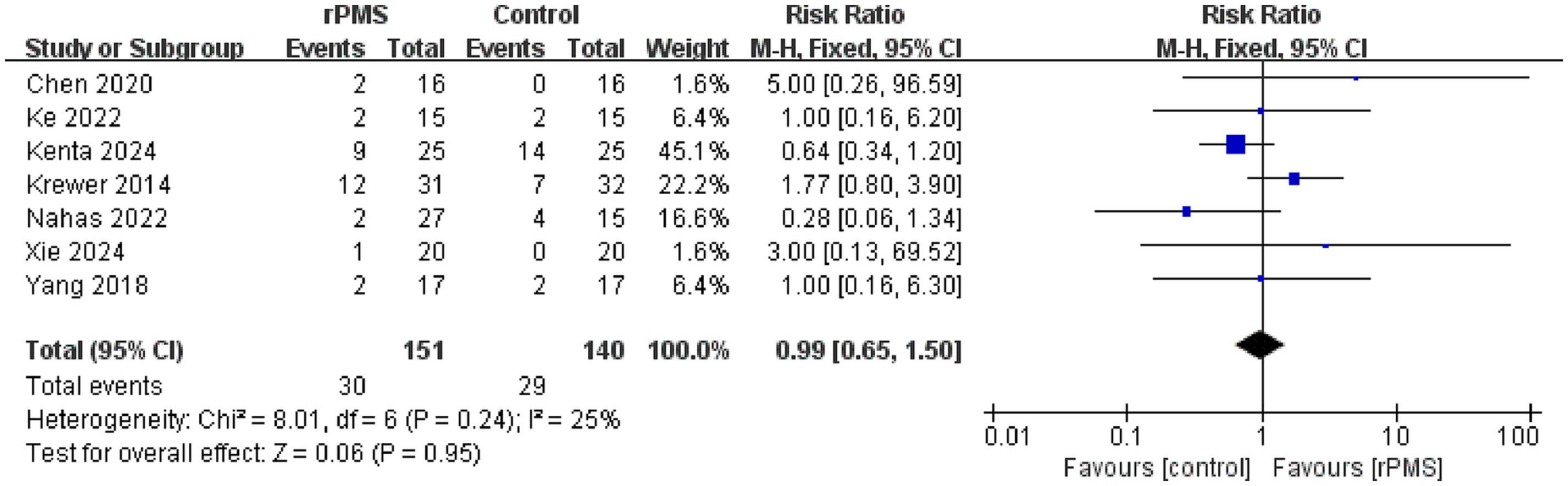
Forest plot of the effects of rPMS on dropout rates in stroke patients.

A total of seven studies (18, 21, 23–27), involving 492 participants, evaluated the effects of rPMS on the dropout rate. Heterogeneity among the included studies was low (*I*^2^ = 25%, *p* = 0.24 > 0.05), and therefore a fixed effects model was used for meta-analysis. The results showed no significant difference between the rPMS group and the control group (RR = 0.99, 95% CI 0.65–1.50, *p* = 0.95) (Figure 11). Four participants in the control group dropped out due to temporary pain, and no adverse events were reported in the rPMS group in the study by Xie et al. (26).

### 3.8 GRADE

According to the GRADE assessment, the overall level of evidence for the effect of rPMS was “High” for the FMA-UE, “Low” for the MAS, “Moderate” for the FIM and MBI, and “Low” for the dropout rate. (Table 2).

**Table 2:** GRADE quality of evidence profile.

Outcome indicator		Number of participants	Heterogeneity		Model of analysis	Group effect value		Estimated value	95% CI	GRADE
			*I* ^2^	*P*		*Z*	*P*			
FMA-UE	Disease stage	334 (9RCT)	78%	<0.0001	Random effects	2.76	0.006	0.69 (SMD)	0.20, 1.17	High
	Stimulation frequency	442 (11RCT)	74%	<0.0001	Random effects	2.91	0.004	0.58 (SMD)	0.19, 0.98	High
	Coil type	301 (8RCT)	75%	0.0002	Random effects	3.19	0.001	0.82 (SMD)	0.31, 1.32	High
	Stimulation duration	414 (10RCT)	76%	<0.0001	Random effects	2.85	0.004	0.62 (SMD)	0.19, 1.05	High
	Stimulation intensity	336 (8RCT)	78%	<0.0001	Random effects	3.11	0.002	0.79 (SMD)	0.29, 1.29	High
MAS		114 (3RCT)	0%	0.97	Fixed effects	1.28	0.20	0.25 (MD)	−0.13,0.63	Low
FIM&MBI		178 (4RCT)	47%	0.13	Fixed effects	4.22	< 0.0001	0.66 (SMD)	0.35,0.96	Moderate
Dropout rate		492 (11RCT)	25%	0.24	Fixed effects	0.06	0.95	0.99 (RR)	0.65,1.50	Low

## 4 Discussion

This study included 12 randomized controlled trials involving a total of 484 participants to systematically evaluate the efficacy of rPMS in treating upper limb dysfunction after stroke. The results demonstrated that rPMS significantly improved upper limb motor function, with particularly notable effects observed in subacute patients receiving treatment protocols characterized by a stimulation frequency of ≤20 Hz, figure-of-eight coils, stimulation durations of 15–20 min, and high intensity. In addition, rPMS effectively enhanced the activities of daily living in patients with upper limb dysfunction. Importantly, rPMS was found to be safe, with no reports of serious adverse effects.

A total of 11 studies (6, 9, 18–22, 24–27) assessed upper limb motor function using the FMA-UE, and the simulated results showed that rPMS could significantly improve upper limb motor function after stroke. A total of eight studies (9, 18, 19, 21, 22, 25–27) revealed that rPMS alone or in combination with physiotherapy and occupational therapy improved upper limb motor function. In addition, two studies (6, 20) suggested that the progress rates of the FMA-UE were significantly different between the two groups. These results are consistent with those of previous systematic reviews (28, 29). Nevertheless, Krewer et al. (24) reported no significant improvement in the motor function of the extensor and flexor muscles of the upper limb after rPMS intervention. There is no statistical evidence suggesting that combining rPMS and transcranial magnetic stimulation (TMS) is more effective than TMS alone in improving motor function (30). Based on the subgroup analysis, these findings may be related to variations in stimulation intensity and frequency, as well as the predominance of chronic-phase patients in the study populations.

The simulated results from the MAS revealed that rPMS had no significant effect on the spasticity of the upper limb. The studies by Chen et al. and Krewe et al. (18, 24) mentioned the use of the Modified Tardieu Scale (MTS) to measure spasticity in patients after stroke, but a meta-analysis could not be completed due to different MTS protocols. Interestingly, Krewe et al. (24) reported a long-term reduction in elbow extensor spasticity after 2 weeks of treatment, but a limited effect on overall upper limb spasticity. In contrast, another study (18) demonstrated an improvement in upper limb spasticity after rPMS, which is inconsistent with our results. Several factors may explain this discrepancy: (1) When focusing on the minimum clinically important difference in a single study, the scores of the MAS may improve by more than 1 point in some patients. However, this statistical difference may not be significant when the effect sizes are combined in a meta-analysis. This suggests that some overall effect sizes, although small yet clinically relevant, may be weakened when simulated. (2) There is variability in patient population and disease severity. There were differences between the studies at baseline in terms of disease duration and the extent of spasticity (baseline MAS score) in the included patients. Patients with higher levels of spasticity are more likely to show improvement with rPMS, while patients with milder spasticity may not show statistically significant changes.

The simulated results showed significant improvements in ADLs compared to the control group, which is consistent with the meta-analysis by Wang et al. (31), indicating that rPMS alone or in combination with rTMS can effectively improve ADLs after stroke. Fine motor movements of the shoulders, elbows, wrists, and fingers are crucial for improving ADL in patients after stroke. Previous studies have shown that, based on improvements in limb motor function in patients after stroke, there are also statistically significant improvements in ADLs (31, 32).

The possible mechanism by which rPMS improves upper limb motor dysfunction after stroke involves multiple neuroplastic regulatory processes. First, rPMS can significantly increase the amplitude of motor evoked potentials via high-frequency stimulation, reduce short-interval intracortical inhibition, and enhance intracortical facilitation. These changes suggest that rPMS can improve the output efficiency of the motor cortex by regulating the balance between inhibitory and excitatory circuits within the cortex (32, 33). For example, Nito et al. reported that 15 min of rPMS at 25 Hz or higher induced an increase in cortical excitability in the relevant area, potentially improving motor output (32). However, this conclusion differs from some subgroup analysis results, possibly due to unidentified underlying factors introducing bias, and therefore requires further research for validation. Second, rPMS treatment can activate neural activity within the superior posterior parietal lobe and premotor cortex, regions closely associated with motor planning and execution. This change may be related to the enhancement of afferent proprioceptive input and the promotion of functional reorganization within the sensorimotor network induced by rPMS, thereby further facilitating motor function recovery (34). Third, rPMS has no significant effect on Hoffmann’s reflex and the maximal M wave, suggesting that its effects are mainly concentrated above the spinal cord level (such as cortical or subcortical structures), rather than directly altering the excitability of spinal motor neurons (32, 33). Finally, animal studies suggest that molecular mechanisms related to rPMS may involve the PHR protein family (e.g., nematode RPM-1), which coordinate the development of motor neural networks by regulating axon termination and synapse formation (35). These underlying mechanisms may provide a molecular basis for the long-term plasticity induced by rPMS (35, 36).

The simulated results of this meta-analysis found that rPMS did not improve the degree of upper limb spasticity in patients after stroke. The possible mechanisms mainly involve three aspects. Firstly, the efficiency of rPMS depends on the frequency, intensity, and location of the stimulation. Low-frequency rPMS (5 Hz) may reduce spasticity by inhibiting spinal reflex arcs. However, if the maintenance of spasticity involves high-frequency abnormal discharges, low-frequency stimulation may not effectively improve spasticity, and the parameters need to be adjusted to match the pathophysiological characteristics (37, 38). In addition, A. Struppler et al. (39) found that rPMS is effective in mild to moderate spasticity but not in severe or fixed muscle contractures, suggesting that structural changes may counteract its neuromodulatory effects. Then, the causes of spasticity are complex and may be related to central sensitization (e.g., imbalance in corticospinal pathway inhibition) or peripheral nerve sensitization (e.g., increased sensitivity of muscle spindle) (38, 40). If spasticity is primarily driven by changes in the peripheral nerve structure (e.g., muscle fibrosis, overactivity of intrafusional *γ* motor neurons), rPMS may have a limited role in regulating the central sensorimotor network (38, 40). Thirdly, the role of rPMS depends, in part, on the activation of proprioceptive afferent fibers (Class Ia fibers) to regulate central motor control. rPMS may not be able to effectively deliver sensory input to the central nervous system, making it difficult to trigger cortical recombination or supraspinal inhibition with peripheral neuropathy or disturbance of sensory conduction (38, 39).

There are limitations in our meta-analysis. First, some outcome measures, such as the MAS, FIM, and MBI, were based on small sample sizes, which limits the stability and reliability of the effect estimates. Second, none of the included studies clearly reported the implementation of blinding, and some data were extracted from images, which may introduce selection bias. Third, despite conducting subgroup analyses, significant heterogeneity remained, suggesting the presence of unidentified potential confounding factors; therefore, the results should be interpreted with caution. Finally, substantial variability in stimulation sites across the studies made it difficult to perform unified subgroup analyses and is likely a major source of the observed heterogeneity. Future high-quality studies focusing on specific target sites are needed to systematically evaluate the therapeutic effects of different stimulation locations, thereby enhancing the accuracy of the conclusions and their clinical applicability.

## 5 Conclusion

The results indicate that rPMS can significantly improve upper limb motor function, ADLs, and self-care abilities in stroke patients, but its effect on spasticity relief is limited. Future research should focus on patients in the subacute phase of stroke and consider using protocols with a stimulation frequency ≤20 Hz, a figure-of-eight coil, a stimulation duration of 15–20 min, and high-intensity stimulation to further verify the efficacy of rPMS. To optimize rPMS treatment protocols, more high-quality studies with larger sample sizes and standardized outcome measures are needed to enhance the reliability and generalizability of the findings.

## Data Availability

The datasets presented in this study can be found in online repositories. The names of the repository/repositories and accession number(s) can be found in the article/Supplementary material.
