# Supplementary material for: Efficacy of repetitive peripheral magnetic stimulation on upper limb motor function after stroke: a systematic review and meta-analysis of randomized controlled trials
Source: Front Neurol. 2025 Sep 18;16:1612490. doi: 10.3389/fneur.2025.1612490 (PMC12488456; doi:10.3389/fneur.2025.1612490)
Supplement: Supplementary file 1 [file Data_Sheet_1.DOCX]

**Table S1.** Search strategies for each database

| **PubMed-810** |
| --- |
| (((((((((((((repeat peripheral magnetic stimulation) OR (peripheral magnetic stimulation)) OR (magnetotherapy)) OR (magnetic field)) OR (magnetism)) OR (magnets)) OR (electromagnets)) OR (electro‐magnets)) OR (PMS)) OR (rPMS)) OR (PrMS)) AND (("Stroke"[Mesh]) OR ((((((((((((((((((((((((((((strokes[Title/Abstract]) OR (cerebrovascular accident[Title/Abstract])) OR (cerebrovascular accidents[Title/Abstract])) OR (cerebral stroke[Title/Abstract])) OR (cerebral strokes[Title/Abstract])) OR (stroke, cerebral[Title/Abstract])) OR (strokes, cerebral[Title/Abstract])) OR (cerebrovascular apoplexy[Title/Abstract])) OR (apoplexy, cerebrovascular[Title/Abstract])) OR (vascular accident, brain[Title/Abstract])) OR (brain vascular accident[Title/Abstract])) OR (brain vascular accidents[Title/Abstract])) OR (vascular accidents, brain[Title/Abstract])) OR (cerebrovascular stroke[Title/Abstract])) OR (cerebrovascular strokes[Title/Abstract])) OR (stroke, cerebrovascular[Title/Abstract])) OR (strokes, cerebrovascular[Title/Abstract])) OR (apoplexy[Title/Abstract])) OR (CVA (cerebrovascular accident[Title/Abstract]))) OR (CVAs (cerebrovascular accident[Title/Abstract]))) OR (stroke, acute[Title/Abstract])) OR (acute stroke[Title/Abstract])) OR (acute strokes[Title/Abstract])) OR (strokes, acute[Title/Abstract])) OR (cerebrovascular accident, acute[Title/Abstract])) OR (acute cerebrovascular accident[Title/Abstract])) OR (acute cerebrovascular accidents[Title/Abstract])) OR (cerebrovascular accidents, acute[Title/Abstract])))) AND ((((((((((((((((((((motor functio[Title/Abstract]) OR (upper limb motor function[Title/Abstract])) OR (extremities, upper[Title/Abstract])) OR (upper extremities[Title/Abstract])) OR (extremity, upper[Title/Abstract])) OR (membrum superius[Title/Abstract])) OR (upper limb[Title/Abstract])) OR (limbs, upper[Title/Abstract])) OR (limb, upper[Title/Abstract])) OR (upper limbs[Title/Abstract])) OR (lower extremity motor function[Title/Abstract])) OR (extremities, lower[Title/Abstract])) OR (lower extremities[Title/Abstract])) OR (extremity, lower[Title/Abstract])) OR (lower limb[Title/Abstract])) OR (limb, lower[Title/Abstract])) OR (limbs, lower[Title/Abstract])) OR (lower limbs[Title/Abstract])) OR (membrum inferius[Title/Abstract])))) |
|  |
| **Embase-6** |
| 1. 'strokes':ab,ti OR 'cerebrovascular accident':ab,ti OR 'cerebrovascular accidents':ab,ti OR 'cerebral stroke':ab,ti OR 'cerebral strokes':ab,ti OR 'stroke, cerebral':ab,ti OR 'strokes, cerebral':ab,ti OR 'cerebrovascular apoplexy':ab,ti OR 'apoplexy, cerebrovascular':ab,ti OR 'vascular accident, brain':ab,ti OR 'brain vascular accident':ab,ti OR 'brain vascular accidents':ab,ti OR 'vascular accidents, brain':ab,ti OR 'cerebrovascular stroke':ab,ti OR 'cerebrovascular strokes':ab,ti OR 'stroke, cerebrovascular':ab,ti OR 'strokes, cerebrovascular':ab,ti OR 'apoplexy':ab,ti OR 'CVA (cerebrovascular accident)':ab,ti OR 'CVAs (cerebrovascular accident)':ab,ti OR 'stroke, acute':ab,ti OR 'acute stroke':ab,ti OR 'acute strokes':ab,ti OR 'strokes, acute':ab,ti OR 'cerebrovascular accident, acute':ab,ti OR 'acute cerebrovascular accident':ab,ti OR 'acute cerebrovascular accidents':ab,ti OR 'cerebrovascular accidents, acute':ab,ti |
| 2. 'motor function':ab,ti OR 'upper limb motor function':ab,ti OR 'upper extremity':ab,ti OR 'extremities, upper':ab,ti OR 'upper extremities':ab,ti OR 'extremity, upper':ab,ti OR 'membrum superius':ab,ti OR 'upper limb':ab,ti OR 'limbs, upper':ab,ti OR 'limb, upper':ab,ti OR 'upper limbs':ab,ti OR 'lower extremity':ab,ti OR 'lower extremity motor function':ab,ti OR 'extremities, lower':ab,ti OR 'lower extremities':ab,ti OR 'extremity, lower':ab,ti OR 'lower limb':ab,ti OR 'limb, lower':ab,ti OR 'limbs, lower':ab,ti OR 'lower limbs':ab,ti OR 'membrum inferius':ab,ti |
| 3. 'repeat peripheral magnetic stimulation':ab,ti OR 'peripheral magnetic stimulation':ab,ti OR 'magnetotherapy':ab,ti OR ' magnetic field':ab,ti OR 'magnetism':ab,ti OR 'magnets':ab,ti OR 'electromagnets':ab,ti OR 'electro‐magnets':ab,ti OR 'PMS':ab,ti OR 'rPMS':ab,ti OR 'PrMS':ab,ti |
| 4. 1 and 2 and 3 |
|  |
| **Web of science-424** |
| 1. strokes OR cerebrovascular accident OR cerebrovascular accidents OR cerebral stroke OR cerebral strokes OR stroke, cerebral OR strokes, cerebral OR cerebrovascular apoplexy OR apoplexy, cerebrovascular OR vascular accident, brain OR brain vascular accident OR brain vascular accidents OR vascular accidents, brain OR cerebrovascular stroke OR cerebrovascular strokes OR stroke, cerebrovascular OR strokes, cerebrovascular OR apoplexy OR CVA (cerebrovascular accident) OR CVAs (cerebrovascular accident) OR stroke, acute OR acute stroke OR acute strokes OR strokes, acute OR cerebrovascular accident, acute OR acute cerebrovascular accident OR acute cerebrovascular accidents OR cerebrovascular accidents, acute |
| 2. motor function OR upper limb motor function OR upper extremity OR extremities, upper OR upper extremities OR extremity, upper OR membrum superius OR upper limb OR limbs, upper OR limb, upper OR upper limbs OR lower extremity OR lower extremity motor function OR extremities, lower OR lower extremities OR extremity, lower OR lower limb OR limb, lower OR limbs, lower OR lower limbs OR membrum inferius |
| 3. repeat peripheral magnetic stimulation OR peripheral magnetic stimulation OR magnetotherapy OR magnetic field OR magnetism OR magnets OR electromagnets OR electro‐magnets OR PMS OR rPMS OR PrMS |
| 4. 1and 2and3 |
|  |
| **Cochranelibrary-26** |
| 1. (strokes):ab,ti,kw OR (cerebrovascular accident):ab,ti,kw OR (cerebrovascular accidents):ab,ti,kw OR (cerebral stroke):ab,ti,kw OR (cerebral strokes):ab,ti,kw OR (stroke, cerebral):ab,ti,kw OR (strokes, cerebral):ab,ti,kw OR (cerebrovascular apoplexy):ab,ti,kw OR (apoplexy, cerebrovascular):ab,ti,kw OR (vascular accident, brain):ab,ti,kw OR (brain vascular accident):ab,ti,kw OR (brain vascular accidents):ab,ti,kw OR (vascular accidents, brain):ab,ti,kw OR (cerebrovascular stroke):ab,ti,kw OR (cerebrovascular strokes):ab,ti,kw OR (stroke, cerebrovascular):ab,ti,kw OR (strokes, cerebrovascular):ab,ti,kw OR (apoplexy):ab,ti,kw OR (CVA (cerebrovascular accident)):ab,ti,kw OR (CVAs (Cerebrovascular Accident)):ab,ti,kw OR (stroke, acute):ab,ti,kw OR (acute stroke):ab,ti,kw OR (acute strokes):ab,ti,kw OR (strokes, acute):ab,ti,kw OR (cerebrovascular accident, acute):ab,ti,kw OR (acute cerebrovascular accident):ab,ti,kw OR (acute cerebrovascular accidents):ab,ti,kw OR (cerebrovascular accidents, acute):ab,ti,kw |
| 2. (motor function):ab,ti,kw OR (upper limb motor function):ab,ti,kw OR (extremities, upper):ab,ti,kw OR (upper extremities):ab,ti,kw OR (extremity, upper):ab,ti,kw OR (membrum superius):ab,ti,kw OR (upper limb):ab,ti,kw OR (limbs, upper):ab,ti,kw OR (limb, upper):ab,ti,kw OR (upper limbs):ab,ti,kw OR (lower extremity motor function):ab,ti,kw OR (extremities, lower):ab,ti,kw OR (lower extremities):ab,ti,kw OR (extremity, lower):ab,ti,kw OR (lower limb):ab,ti,kw OR (limb, lower):ab,ti,kw OR (limbs, lower):ab,ti,kw OR (lower limbs):ab,ti,kw OR (membrum inferius):ab,ti,kw |
| 3. (repeat peripheral magnetic stimulation):ab,ti,kw OR (peripheral magnetic stimulation):ab,ti,kw OR (magnetotherapy):ab,ti,kw OR (magnetic field):ab,ti,kw OR (magnetism):ab,ti,kw OR (magnets):ab,ti,kw OR (electromagnets):ab,ti,kw OR ( electro‐magnets):ab,ti,kw OR (PMS):ab,ti,kw OR (rPMS):ab,ti,kw OR (PrMS):ab,ti,kw |
| 4. 1 and 2 and 3 |
|  |
| **CNKI-2** |
| （主题：卒中后上肢功能障碍）OR（主题：脑中风后上肢运动障碍）OR（主题：脑卒中上肢运动功能）OR（主题：中风后上肢康复）OR（主题：脑卒中后运动功能恢复）AND（主题：重复性外周磁刺激）OR（主题：rPMS）OR（主题：高频重复性外周磁刺激）OR（主题：HF-rPMS） |
